# Explaining regional variations in health care utilization between Swiss cantons using panel econometric models

**DOI:** 10.1186/1472-6963-12-62

**Published:** 2012-03-13

**Authors:** Paul A Camenzind

**Affiliations:** 1 Swiss Health Observatory and University of Neuchâtel, Espace de l'Europe 10, 2010 Neuchâtel, Switzerland

## Abstract

**Background:**

In spite of a detailed and nation-wide legislation frame, there exist large cantonal disparities in consumed quantities of health care services in Switzerland. In this study, the most important factors of influence causing these regional disparities are determined. The findings can also be productive for discussing the containment of health care consumption in other countries.

**Methods:**

Based on the literature, relevant factors that cause geographic disparities of quantities and costs in western health care systems are identified. Using a selected set of these factors, individual panel econometric models are calculated to explain the variation of the utilization in each of the six largest health care service groups (general practitioners, specialist doctors, hospital inpatient, hospital outpatient, medication, and nursing homes) in Swiss mandatory health insurance (MHI). The main data source is 'Datenpool santésuisse', a database of Swiss health insurers.

**Results:**

For all six health care service groups, significant factors influencing the utilization frequency over time and across cantons are found. A greater supply of service providers tends to have strong interrelations with per capita consumption of MHI services. On the demand side, older populations and higher population densities represent the clearest driving factors.

**Conclusions:**

Strategies to contain consumption and costs in health care should include several elements. In the federalist Swiss system, the structure of regional health care supply seems to generate significant effects. However, the extent of driving factors on the demand side (e.g., social deprivation) or financing instruments (e.g., high deductibles) should also be considered.

## Background

**Figure 1:**
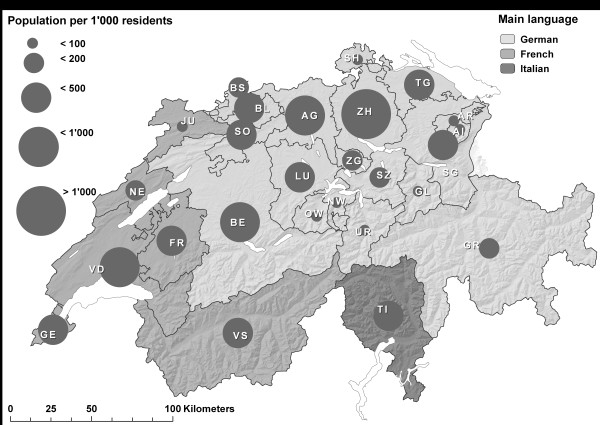
**Population size and main language of the 26 Swiss cantons^1^, 2009**.

Switzerland is a small, western European country with a population in 2009 of 7.8 million persons who live on a total area of 41,000 km^2^. The country consists of 26 cantons, which differ thoroughly in terms of area, number of inhabitants, population density, socio-economic situation, and language (see Figure 1).

1) See acronyms, full cantonal names, and more cantonal characteristics in Appendix, Table 4.

The responsibility of government is divided into three state levels: the central government (i.e., 'confederation'), the 26 cantons, and approximately 2,600 municipalities. The three levels also intervene in the Swiss health care system, which is normally characterized as a 'system of regulated competition' [1]. Briefly said, 'regulated competition' implies competition between health care market players wherever competition seems to generate better outcomes than regulation by state authorities. Some important characteristics of health care market players are described in Table 1. In particular, the outpatient health service providers in Switzerland operate mainly on a private basis, while private- and public-owned institutions are responsible for the provision of inpatient services.

**Table 1:** Swiss 'market' for mandatory health insurance (MHI), 2007

Main actors/components	Number of actors	Number of beds	Owner-ship	MHI costs (billion CHF)	General remarks
MHI companies	87	-	private	-	MHI companies authorized by the confederation and obliged to contract with service providers ('contract obligation')

General practitioners in private practices (GP)	5,915	-	private	2.0 (10.6%)	Calculated full-time employees for general practitioners, pediatrics and gynecologists in private practices (MHI services)

Specialist doctors in private practices	3,244	-	private	1.9 (10.2%)	Calculated full-time employees for specialized physicians in private practices (MHI services)

Hospitals inpatient	321	41,910	public & private	4.8 (26.3%)	General hospitals and specialized clinics for psychiatry and rehabilitation (MHI services)

Hospitals outpatient	> 130	-	public & private	2.8 (14.9%)	General hospitals (130) normally supply outpatient care; but this is not known for all the specialized clinics (181)

Drugs	1,700/~4,000	-	private	3.6 (19.5%)	Number of pharmacies: 1,700; number of self dispensing physicians: ~4,000 (MHI services)

Long-term care homes	1,509	87,960	public & private	1.6 (8.7%)	Nursing homes (without homes for disabled, for addicts and persons with psychosocial problems, MHI services)

Total of MHI services delivered	-	-	-	18.5 (100%)	Other providers account for the rest of CHF 1.8 billion (9.8%); MHI administration costs and cost participations are not included here

Sources: [2-5], own calculations.

Although the regulatory responsibilities and public funding of Swiss health care are shared by three state levels, the 26 cantons are mostly responsible for the implementation of health policy. This includes the mission to guarantee sufficient supply of inpatient and outpatient services for their populations and to contribute the largest part of the state payments.

The confederation, on the other hand, has more legislative functions in the area of MHI and supervising functions for the MHI insurance market. It is responsible for the nation-wide health care legislation, specifically the definition of the package of services covered by the MHI. Other federal responsibilities include the setting of standards of education and training of the health care personnel, populations' health protection, surveillance and management of transferable diseases, and health sciences and research.

Finally, the municipalities see their most important role in carrying out the tasks handed over to them by the cantons. In most cases, these tasks involve the operation and funding of providers either in outpatient or inpatient long-term care (i.e., care at home and nursing homes).

The two important characteristics of the Swiss health care system mentioned above, regulated competition and federalist structure, are intensively discussed in Swiss health politics. In spite of clear advantages such as efficiency gains and proximity to the local populations and their needs, there are also major obstacles to overcome. Therefore, the system has an enormous complexity, and good governance in Swiss health care seems difficult [6]. Certain amounts of incoherence, twin-track processes, and obstructions in health care regulation beyond the national or cantonal level have to be accepted.

Empirically, it is not consistently proven yet whether, all in all, such a system leads to higher - or lower - health care costs than in other comparable countries [7]. However, it is clear that regulated competition and strong federalism result in large disparities in health care utilization and costs across geographical areas (see Appendix, Table 4).

Important work to identify reasons for growing and disparate health care expenditures - and utilization - in western countries started about 50 years ago [8]. In this research, tax-financed health care systems provide 'free' health care services to their users, and therefore higher shares of public financing lead to higher spending on health in such societies. In the 'model of unbalanced growth' [9], the nominal wages in the health care sector must be increased, even without progress in productivity. This causes more spending on health care as the economy grows. In later studies [10,11], combinations of higher national income and greater share of public financing were suspected to have driving effects on both the demand and the supply of health care.

Many more models that can potentially determine the influence factors on health care costs and utilization have been developed and tested [12,13]. As mentioned earlier by internationally-recognized researchers [12,14,15], there is still no widely accepted theoretical base to explain international or regional disparities in level and growth of health care spending [7]. This is due to the great complexity and heterogeneity of the organization and the financing of health care systems, the multiplicity of actors involved, and the specific nature of 'health' as an economic good [16].

The available literature of interest continues to focus on finding factors to explain international or intra-national disparities in levels and growth of health care costs and related components. One actual literature review about the subject [7] finds rising incomes, ageing population (i.e., proximity to death), technological progress, and territorial decentralization as the main health care cost drivers in western economies.

A Swiss study on the subject explains the regional disparities in level and growth of health care costs across the country by applying cross-sectional regressions [17]. This study identifies significant correlations between higher health care costs and stronger social disintegration, more unemployment, higher concentration of physicians, more specialized clinics, and a Latin-speaking (i.e., French- and Italian-speaking; see Figure 1) population. Later studies using panel econometric approaches [18,19] find significant influences due to higher income levels, older populations, higher mortality rates, denser populations, and a time trend.

As 'new' significant factors in another Swiss study using cross-sectional regressions [20], higher numbers of women in the population and more pharmacies per capita are detected. In a more recent study [21] applying exactly the same methods but with more recent data [18], a greater number of small children in the population and higher prices for MHI services are identified as significant explanatory factors for cantonal cost differences. Also using econometric panel models, the most recent study consulted [22] indicates significant cost driving effects of a higher number of drug-dispensing physicians and more foreigners in the population, while more managed care models tend to contain health costs.

Most of these studies that compare health expenditures on the international level try to work with the largest possible aggregates of costs [7,12]. This seems reasonable because it is the best way to overcome the problem of enormous differences in the structure of the provision and the financing of health care services in different (western) countries.

Furthermore, some studies on regional variations in health care costs in Switzerland [17,18,21,22] put much effort into creating the broadest possible cost aggregates. Other studies [19,20,23] argue that higher aggregated cost units tend to create more reversal influences. The higher aggregates often compensate each other, and therefore the existing disparities are more likely to 'disappear' statistically. The conclusion drawn from this fact for this work is that the factors responsible for cost differences should be scrutinized at the highest level of detail.

The research interest in this paper follows the cited literature: it aims to empirically consolidate the most important factors for varying health care utilization on the supply side, the demand side, the financing side, and the political-cultural side of the Swiss health care system [18,23]. On the supply side, it is the link between supply and utilization of health care services [24-26] that can be examined in particular. As most MHI services in Switzerland are remunerated in a fee-for-service system in the observed time period and most of the service providers have clear incentives to bring their income to their targeted level [26-28], it is reasonable to suppose that higher densities of general practitioners (GP), specialists, hospital beds, and hospital outpatient care supply lead to higher consumption levels in the four corresponding service groups as well as in outpatient medication.

On the demand side, it will be tested whether higher proportions of older people with greater morbidity and mortality [29], larger population densities indicating urban regions with fewer social barriers to seeing health professionals rather quickly [30], and higher unemployment associated with poorer health status [31] indeed cause driving effects in the use of MHI health services. In addition, it is also analyzed whether higher average income - normally related with better health status, better health behavior, and more supplemental (private) health insurance [21] - is associated with a decrease in the utilization of MHI health services.

Concerning financing of health care, this paper attempts to prove empirically whether high deductibles - the standard deductible in MHI is Swiss francs (CHF) 300 by default, but it can be raised to CHF 2,500 in favor of a limited premium reduction and health commodities consumed within one year, and up to these amounts have to be paid out of pocket by policy holders - and alternative health insurance plans - containing institutional requirements and financial incentives such as gate keeping, limited access to providers, and capitation schemes - favor the utilization of GP [28,29] and outpatient services, a cost-conscious consumption of drugs [32], and a reduction of the use of specialists, outpatient hospitals, inpatient hospitals, and nursing homes.

With regard to political-cultural variables, the Latin-speaking part of Switzerland is often said to gravitate toward an increased utilization of services of specialists, inpatient and outpatient hospitals, and higher drug consumption, but with less frequent use of GP and nursing home services [17,18,21]. Thus, it is empirically tested whether such cultural differences could be responsible for this deviating consumption behavior.

## Methods

### Data

The main data source for the six dependent variables (see Appendix, Table 5) is 'Datenpool santésuisse' (DPS) of the Swiss Association of Health Insurers [33]. DPS has been in existence since 1997, but the complete sets of quantity (and cost) indicators of MHI services used here are only recorded every year starting in 2000. DPS allows for the relating of MHI costs for age and gender groups of the resident (cantonal) population and the various groups of services (which can be located in the same or another canton). DPS is not available to the general public. But on request, the Swiss Association of Health Insurers can put the data at researchers' disposal.

In addition to the costs, DPS also details the quantities of services delivered: the number of consultations and home visits (i.e., 'basic services') of GP and specialists, the number of consultations in outpatient hospitals, the number of hospital days, and the number of days of stay in nursing homes and cost volumes for drugs. Quantities - and cost volumes for drugs - are calculated per capita (i.e., per head of the cantonal population). To get these numbers, the average resident population according to population statistics (ESPOP) [34] of the Swiss Federal Statistical Office (FSO) is used.

DPS and ESPOP statistics are also used to calculate several of the independent variables (see Appendix, Table 5): the densities of GP (GRU) and specialists (SPZ), the percentage of outpatient hospital MHI costs compared to the total outpatient MHI costs (PAM), the proportion of people with deductibles higher than CHF 300 compared to the total number of insured people in the MHI (FRA), the share of alternative MHI plans compared to the total number of MHI insurance contracts (MOD), and the proportion of the population over 65 years of age compared to the total population (ALT65). Additionally, the share of the population over 85 years of age (ALT85) is calculated to explain nursing home utilization [19].

ESPOP is also employed as an 'auxiliary variable' with three other independent variables: the FSO data sources 'Medical Statistics of Hospitals' (MSH) [3] to calculate the density of hospital beds (BED), 'Areal Statistics' [35] to calculate the population density per canton (POP), and 'Statistics of Registered Unemployment' [36] of the State Secretariat for Economic Affairs (SECO) to calculate the cantonal unemployment rates (ALQ). The cantonal incomes per capita (VEL) come from the 'Statistics on National Accounts' [37]. Finally, the proportion of the Latin-speaking resident population as a percentage of the resident population with 'major language non-German' (LAT) from the federal population census 2000 [38] is calculated.

### Data analysis

As explained in the background section, an approach of 'disaggregated identification of influence factors' is emphasized in this work. Therefore, individual analyses on the cost 'sub-component quantity' of the 'subgroups of MHI services' (general practitioners, specialist doctors, hospital inpatient, hospital outpatient, medication and nursing homes) are carried out.

'Quantity' delineates the number of consultations and home visits (basic services) per capita and year of GP (AZG) and specialists (AZS), the number of consultations per capita and year in outpatient hospitals (AMB), the number of days per capita and year in hospital (HOS), and the length of stay in days per capita and year in nursing homes (SOM). Since there is no quantity indicator available for consumed drugs, cost volumes per capita and year (MED) are used.

As suggested by the literature, supply-side, demand-side, financing, and political-cultural variables create the influencing factors (independent variables) of the models. Thus, indicators for the four most important suppliers of health care are used (see Table 1). These include the 'density of GP' (GRU), the 'density of specialists' (SPZ), the 'density of hospital beds' (BED), and 'higher proportions of outpatient hospital care' (AMB). The four most cited indicators on the demand side in the literature, more old people (ALT65), higher average incomes (VEL), larger population densities (POP), and social deprivation - operationalized as higher rates of unemployment (ALQ) in the canton - are kept.

From the two available financing variables, both the share of low deductibles (FRA) and the share of alternative health insurance plans (MOD) are retained in the models, as well as the share of Latin-speaking population (LAT) as political-cultural variable. Finally, a trend variable (TRD) that records medical-technical progress and general tendencies for every group of services that are not yet contained in the other explaining factors is added.

With the goal of a simultaneous explanation of the regional variations in level and evolution of MHI services, panel econometric regressions that combine longitudinal and cross-sectional observations are chosen. The indices 'i' for 26 cantons and 't' for eight years give expression to this combination in Formula (1).

(1) $${\text{Y}}_{\text{it}}=\;f\;\left(\text{GR}{\text{U}}_{\text{it}},\text{ SP}{\text{Z}}_{\text{it}},\text{ BE}{\text{D}}_{\text{it}},\text{ PA}{\text{M}}_{\text{it}},\text{ ALT65}/\text{8}{\text{5}}_{\text{it}},\text{ PO}{\text{P}}_{\text{it}},\text{ AL}{\text{Q}}_{\text{it}},\text{ VE}{\text{L}}_{\text{it}},\text{ FR}{\text{A}}_{\text{it}},\text{ MO}{\text{D}}_{\text{it}},\text{ LA}{\text{T}}_{\text{i}},\text{ TR}{\text{D}}_{\text{t}}\right)$$

where: Y_it _= AZG_it _or AZS_it _or HOS_it _or AMB_it _or MED_it _or SOM_it_;

and: i = 1,2, ..., 26; t = 2000, 2001, ..., 2007.

The statistical examination of the six dependent variables Y_it _shows that they are all in continuous form, normally distributed, and do not contain any outliers, so the use of a parametric linear model is appropriate [39]. Pooled-regression models (PRM) for panel data, fixed-effects models (FEM), and random-effects models (REM) can be applied for this purpose. However, compared to FEM and REM, the results of PRM are not efficient.

The Hausman test is used to check whether - given the existence of unbiased estimators - not only FEM but also REM are consistent. FEM have the limitation that they can only take into account the variation within the groups of observed individuals. FEM estimate individual cantonal parameters in addition to those of the explanatory variables. Therefore, with limited observations, and to retain as much explanatory power as possible, it makes sense to estimate FEM by a within transform of the model (i.e., mean-differencing every variable with respect to its cantonal mean). With the corresponding 'FEM-within-routine' [39], all coefficients are estimated.

REM make use of variances both within groups and between groups of observations. More information is used in this case, and so REM are generally more efficient than FEM. To concretely estimate such (consistent) REM, routines according to the generalized least squares procedure are applied. Finally, four FEM (for AZG, AZS, AMB and MED) and two REM (for HOS and SOM) are calculated. Moreover, F-tests (in FEM) and Wald tests (in REM) are applied to verify individual and joint linearity of the estimated parameters. All calculations and tests were performed with STATA11^® ^software.

## Results

Participating in DPS is optional for Swiss health insurance companies, and so its coverage in 2007 (2000) applies to 98% (92%) of all insured people. To estimate complete values, the data are extrapolated using the statistics of the 'Common Institution under the Federal Health Insurance Act' [40]. The 'Common Institution' runs the risk adjustment system in the MHI, so its highly-aggregated indicators cover all insured persons (i.e., the entire Swiss population). The extrapolation assumes that the missing 2% (8%) of persons in DPS have the same cost structure as the 98% (92%) of insured people who are included in DPS in 2007 (2000). All calculations presented hereafter are based on the extrapolated values of DPS.

Table 2 contains different indicators to describe the cantonal disparities in level and growth of the six dependent variables, which cover more than 90% of MHI health services (see Table 1). The average number of basic services of GP per capita and year (AZG) between 2000 and 2007 is 3.7, and it varies between 2.3 contacts (canton Geneva GE and Jura JU) and 4.7 contacts (Glarus GL); this corresponds to an EQ-ratio of around 1.6. The same indicators for specialists (AZS) are 1.2 (mean) and show fluctuations between 0.7 contacts (Nidwalden NW, Obwalden OW and Uri UR) and 2.1 contacts in Basel-Stadt BS; the EQ for specialist services is greater here at 3.2, indicating larger disparities between cantons than for GP.

**Table 2:** Dependent variables: levels^1) ^and trends^2)^, 2000-2007

MHI service groups: per capita utilization	n (CAN-TON^3)^)	T (YEAR)	N (OBS)	MEAN^4)^	STD^4)^	MIN^4)^	MAX^4)^	EQ^4)^	**Δ% 2000-2007**^**2**^**)**
**General practitioners: basic services (AZG)**	26	8	208	3.7	0.5	2.9	4.7	1.6	-0.2%

**Specialist doctors: basic services (AZS)**	26	8	208	1.2	0.3	0.7	2.1	3.2	-0.9%

**Hospital inpatient: hospital days (HOS)**	26	8	208	1.9	0.5	1.3	3.5	2.7	-0.5%

**Hospital outpatient: consultations (AMB)**	26	8	208	0.9	0.5	0.6	2.7	4.5	7.1%

**Drugs outpatient:costs (MED)**	26	8	208	420	89	287	572	2.0	3.2%

**Nursing homes:days of stay (SOM)**	26	8	208	3.4	1.0	2.2	5.9	2.6	6.6%

Source: [33], own calculations.

1) Average absolute numbers of per capita services, days and costs between 2000 and 2007.

2) Average annual growth rates in % of per capita services, days and costs between 2000 and 2007 (Δ% 2000-2007).

3) See Figure 1 and full names and more characteristics of cantons in Appendix, Table 4.

4) MEAN = arithmetical mean; STD = standard deviation; MIN = minimal value; MAX = maximal value; EQ = extremal quotient.

Hospital days per capita and year (HOS) are 1.9 days per 1,000 persons. The cantonal variation ranges from 1.3 days (Nidwalden NW, Obwalden OW and Zug ZG) to 3.5 days (Basel-Stadt BS), which results in an EQ-ratio of 2.7. The consultations in outpatient hospitals (AMB) are at 0.9 per person per year. They fluctuate between 0.6 days (Schwyz SZ) and 2.7 days (Basel-Stadt BS), and the ratio between these two extreme values is actually 4.5. This is the largest inter-cantonal variation across the six dependent variables.

The average annual costs per person for outpatient medication (MED) are at CHF 420 and range from CHF 287 (Appenzell Innerrhoden AI) to CHF 572 (Basel-Stadt BS), with an EQ-ratio of 2.0. Finally, there is an average of 3.4 nursing home days per capita and year (SOM) remunerated in the MHI; this number varies between 2.2 (Valais VS) and 5.9 (Appenzell Ausserhoden AR). Here, the result is an EQ-ratio of 2.6.

The last indicator in Table 2 describes the general cantonal trends in the six variables during the observed period of eight years (Δ% 2000-2007). The tendencies for the utilization of basic services of GP per capita (AZG; -0.2% for the whole country) between 2000 and 2007 are non-uniform: one half of the cantons show rising trends, and the other half show decreasing trends for this indicator. The result is also geographically non-uniform for the utilization of specialists (AZS; -0.9%), but particularly large cantons (see Figure 1) show decreasing trends. For the number of hospital days per capita and year (HOS; -0.5%), some more cantons with decreasing trends can be found as well.

The other three dependent variables, the consultations in outpatient hospitals (AMB; +7.1%), the costs for outpatient medication (MED; +3.2%), and the number of nursing home days (SOM; +6.6%), show expanding tendencies across all cantons. There is only one exception from this general trend: in canton Basel-Stadt (BS), the number of consultations in outpatient hospitals per capita and year (AMB) declined from 3.1 in 2000 to 2.5 in 2007 (-3.0% annually).

Table 3 shows the results of the multivariate estimates for the six explanatory models. As mentioned above, four fixed-effects models for the dependent variables AZG, AZS, AMB, MED (i.e., outpatient services) and two random-effects models for the dependent variables HOS and SOM (i.e., inpatient services) are calculated.

**Table 3:** Multivariate estimation results

Estimation technique	FEM	FEM	REM	FEM	FEM	REM
	**General. practi-tioners basic services AZG**	**Specialist doctors basic services AZS**	**Hospital inpatient hospital days HOS**	**Hospital outpatient consul-tations AMB**	**Drugs outpatient costs MED**	**Nursing homes days of stay SOM**

Density of general practitioners **GRU**	0.042*** (0.0036)	**0.002 **(0.0022)	**-0.0003 **(0.0036)	-0.017*** (0.0046)	1.379*** (0.4920)	0.020** (0.0081)

Density of specialist doctors **SPZ**	0.013*** (0.0038)	0.020*** (0.0024)	**0.001 **(0.0038)	**-0.004 **(0.0049)	1.322** (0.5218)	-0.026*** (0.0086)

Density of hospital beds **BED**	-0.0001*** (0.0002)	**-0.00007 **(0.0001)	**0.0003 **(0.0002)	**-0.00001 **(0.0002)	**0.022 **(0.0257)	0.001** (0.0004)

Share of hospital outpatient costs **PAM**	0.012** (0.0057)	**0.004 **(0.0036)	**-0.0001 **(0.0081)	0.059*** (0.0074)	**-0.935 **(0.7881)	-0.060*** (0.0159)

Population 65+/85+ **ALT65/ALT85**^1)^	**0.004 **(0.0033)	**0.002 **(0.0021)	0.011*** (0.0026)	0.020*** (0.0043)	**-0.644 **(0.4610)	0.175***^1) ^(0.0218)

Population density **POP**	0.259*** (0.0772)	0.116** (0.0486)	0.013** (0.0061)	**0.209 **(0.1164)	-22.629** (10.6982)	**-0.025 **(0.0159)

Unemployment rate **ALQ**	-0.019*** (0.0031)	-0.015*** (0.0020)	0.014*** (0.0040)	-0.023*** (0.0047)	1.348*** (0.4308)	0.020** (0.0083)

Average cantonal income **VEL**	0.390*** (0.1416)	**0.083 **(0.089)	**0.128 **(0.1440)	**-0.031 **(0.1847)	**14.903 **(19.6131)	0.716** (0.3266)

Share of higher deductibles **FRA**	0.001*** (0.0004)	0.001*** (0.0002)	**-0.00004 **(0.0004)	0.001** (0.0005)	-0.132*** (0.0487)	0.003*** (0.0009)

Alternative MHI-plans **MOD**	-0.001** (0.0004)	**0.0003 **(0.0002)	**0.0002 **(0.0005)	**-0.001 **(0.0005)	-0.213*** (0.0511)	**0.0000 **(0.0001)

Share of Latin-speaking pop. **LAT**	omitted	omitted	**0.0001 **(0.0002)	omitted	omitted	-0.002*** (0.0004)

Trend variable **TRD**	**-0.016 **(0.0105)	-0.021*** (0.0066)	-0.047*** (0.0124)	**0.022 **(0.0137)	15.121*** (1.4550)	0.153*** (0.0255)

Source: own calculations.

Standard error in parenthesis. *** = significant at 0.01%, ** = significant at 0.05%; omitted = FEM can only estimate coefficients for variables that vary over time.

1) To estimate the utilization of nursing homes, the population 85 years and older is used.

The cantonal and time-related variations of the utilization of GP services per capita (AZG) are significant and positively linked with higher densities of general practitioners (GRU) and specialists (SPZ), a larger share of outpatient hospital services (PAM), a greater population density (POP), a higher average cantonal income (VEL), and a larger share of high deductibles (FRA). However, there is a negative relationship between GP utilization and a greater hospital bed density (BED), a higher unemployment rate (ALQ), and more alternative MHI insurance plans (MOD).

The utilization of specialist services per capita (AZS) is linked with a higher density of specialists (SPZ), a higher population density (POP), and a larger share of high deductibles (FRA). Otherwise, lower unemployment rates (ALQ) and the trend variable (TRD) accompany reduced utilization of specialists.

The number of (inpatient) hospital days per head of population (HOS) shows to be 'driven' by an older population (ALT65), a higher population density (POP), and higher unemployment (ALQ). The trend variable (TRD) expresses the general downward trend in the per capita utilization of inpatient hospitals.

For differences in the quantities of outpatient hospitals utilization (AMB), the estimates show driving effects of a larger share of outpatient hospital services (PAM), an older population (ALT65), and a larger share of high deductibles (FRA). Slowing effects come from a greater supply of GP (GRU) and a higher unemployment rate (ALQ), while all other factors prove to be insignificant.

The variation in drug costs per head of population (MED) is highly significant and positively associated with a higher density of GP (GRU) and specialists (SPZ), the cantonal unemployment rate (ALQ), and the trend variable (TRD). A greater population density (POP), a larger share of high deductibles (FRA), and a larger proportion of alternative MHI plans (MOD) are significant and negatively related with drug costs per capita (MED).

Finally, the following factors are shown to have significant positive effects on the per capita differences in nursing home utilization (SOM): a higher density of GP (GRU) and of hospital beds (BED), a larger share of population older than 85 years (ALT85), a higher unemployment rate (ALQ), a higher average cantonal income (VEL), a larger share of high deductibles (FRA), and the general trend (TRD). On the other hand, slowing effects on utilization of nursing homes per capita (SOM) seem to result from a higher (potential) supply of specialists (SPZ), a greater share of hospital outpatient services (PAM), and a mainly Latin-speaking environment and pattern of use (LAT).

## Discussion

The results' section described how the interplay of the independent variables influences the utilization of every individual group of MHI services. Due to having used exactly the same set of independent variables for all six models, the perspective can now be changed: the influence of every independent variable on the six groups of MHI services can be discussed simultaneously by comparing them across these groups.

Higher densities of general practitioners (GRU) tend to reinforce the per capita use of their services, the use of drugs, and the number of days per capita in nursing homes, the lattermost relation expressing the intense collaboration of GP with nursing homes in Switzerland. A higher potential supply of specialists (SPZ) also drives - beside the use of their own services - the utilization of GP services and the consumption of drugs. More hospital beds (BED) seem to reinforce - more than the utilization of their own services - the use of nursing homes, an expression of the close collaboration of these two groups of inpatient health service providers. Finally, a higher share of hospital outpatient services on total outpatient services (PAM) goes hand in hand with more intense per capita utilization of such services, including GP services.

All four supply-side variables each show one significant slowing influence factor. More GP (GRU) tend to slow down the utilization of - relatively expensive - outpatient hospitals. This could be an important finding in terms of stabilizing health care costs. More specialists (SPZ) and a higher share of hospital outpatient services (PAM) curtail the utilization of nursing homes. These results are positive in the sense that a growing number of older people in Switzerland are trying to avoid institutionalization in homes as long as possible. In an isolated consideration of MHI costs only, this behavior of older people tends to have rather cost driving effects: most of the costs of specialist and outpatient hospital services are paid by MHI, whereas a large part of nursing home financing comes from outside the MHI - primarily out-of-pocket payments. More hospital beds (BED) mean less frequent utilization of GP. The function of GP warranting health service supply in remote areas of the country - without hospitals - could explain this negative association.

Caution is warranted in directly concluding supplier inducement from the association between higher population-physician or population-hospital beds ratios and more extensive utilization of medical services [24-26,28]. This relationship could also reflect an effect of lower prices on patient demand, a supply response to variation in health status, or improved availability. Conversely, in health care systems, where service providers are mainly remunerated by fee-for-service schemes and have clear incentives to bring their income to a targeted level, the existence of over-supply and supplier inducement is probable.

Moreover, these presented effects of the supply-side factors might also be an expression of two different types of cantonal health delivery schemes, as they were described in a former Swiss study using qualitative approaches [41]. On one hand, they may show a 'center-type scheme', in which high - inpatient and outpatient - hospital and specialist density and use accompany intensive supply and utilization of hospital and specialist services. On the other hand, they may reflect the 'peripheral-type scheme' of a cantonal health system, which is more focused on primary care and nursing homes, while supply and utilization of specialists and hospital services are restrained.

Driving effects on the use of health care providers for all four tested factors on the demand side are found. Higher age (ALT65/ALT85) is responsible for higher utilization of inpatient and outpatient hospitals and nursing homes. This result makes sense in the nursing home branch, but the discussion of the relationship between high age and hospital utilization could be enlarged by arguments coming from literature about 'proximity to death' [42]. Higher population densities (POP) have driving effects on the utilization of both types of physicians and on inpatient hospitals creating fewer social barriers in urban areas to seeing a health professional quickly. High unemployment (ALQ) has driving effects on the consumption of drugs and on more frequent/longer stays in hospitals and nursing homes. Finally, a higher average cantonal income (VEL) is significantly correlated with higher levels of utilization of per capita GP services and nursing homes. This is also the case for specialists, hospital inpatient services, and drug costs, although these interrelations are not statistically significant.

Among these demand-side variables, the unemployment rate (ALQ) shows significant slowing effects with respect to per capita outpatient consultations with GP, specialists, and hospitals. The financial difficulties of unemployed persons could explain this finding and merits special attention. It could suggest that unemployed people avoid outpatient treatments and compensate this with greater drug consumption and more/longer unavoidable stays in hospitals or nursing homes. An undersupply of such groups of vulnerable people in the outpatient sector [43] can have serious medical, social, and financial consequences.

The negative association between population density (POP) and outpatient medication costs per capita was unexpected. An explanation could be that a higher number of hospital activities in urban areas partially substitute provision of drugs delivered by pharmacies and physicians - contained in the indicator, MED. It could also indicate that people in urban areas show a tendency to pay more out of pocket for drugs, particularly in combination with more high deductibles.

Regarding the two indicators of health services financing, one would expect slowing effects from higher deductibles (FRA) and more alternative MHI plans (MOD) on all groups of health services, except on GP with their coordination functions in alternative MHI plans [44]. However, the results in Table 3 show that more deductibles actually go along with more intense utilization of GP, specialists, hospital outpatient services, and nursing homes. One explanation for this result could be the skewed distribution of health care costs: 30% of all insured people cause approximately 80% of total MHI costs in Switzerland [45]. As a result, the potential to reduce unnecessary use of health care services by financial incentives like higher deductibles remains limited [46]. Exceptions are outpatient drugs where more deductibles and more alternative health insurance plans seem to be able to lower the medication costs per capita (MED). Moreover, the negative association between more alternative health insurance plans and GP utilization does not confirm the assumption of higher utilization because of the coordination function of GP in such plans. This could connect with the limitation of the model that *only *frequencies of contacts are measured in the models. Better coordination by GP does not necessarily mean more frequent use of the services of this group of providers.

The 'political-cultural' variable, the share of Latin-speaking people (LAT), reveals only one significant effect in the estimation models, namely a reduced utilization of nursing homes. The data used here cannot explain whether this is due to epidemiological reasons, substitution of long-term care with more informal care at home, or more professional outpatient (or even hospital) long-term care [47].

The trend variables (TRD) can explain additional variance of the dependent variables in the six models. The accelerations observed for drugs, nursing homes, and hospital outpatient services (not significant) are consistent with general expectations (see Table 2). The same is true for the declining trends of inpatient hospital utilization and per capita use of specialists and GP (not significant). Whereas declining trends for GP and inpatient hospitals are broadly expected in Switzerland [19], most people would not expect such a reduction for specialist doctors who have constantly rising costs, but this finding can be further explained with a limitation in the dependent 'quantity' variables. As one can only count the number of consultations in the outpatient sector and the number of days in the inpatient sector, changes in medical practices [48] that occur across cantons and over time cannot be seen. Thus, for specialist doctors in Switzerland there exists a trend toward fewer consultations per capita during the observed time period (see Table 2).

## Conclusions

The findings of this paper confirm that consumption and cost-containment strategies in health care should integrate several supply-side, demand-side, and financing elements [23]. First, the cantonal structure of the health care supply system turns out to be a crucial element. High densities of health care suppliers result - at least in their combination with fee-for-service remuneration, target incomes, and contracting obligation for insurers - in more intense utilization per capita of most groups of health care services and in greater drug consumption [28]. Whether or not this happens through supplier-induced demand, health policy authorities should be made aware of this fact.

Among these supply-side elements, special attention should be paid to the growing amounts of relatively expensive hospital outpatient services [49]. The results in the models show that higher densities of GP could have relieving effects on hospital outpatient use, and so a geographically well-distributed supply of primary care services is important. Moreover, it seems that a greater supply of specialized and relatively costly outpatient health care services reduces the intensity of nursing home utilization [50]. Health politicians should be aware that what is beneficial for older patients - who normally try to avoid institutionalization with its relatively large out-of-pocket and relatively small MHI funding in Switzerland as long as possible - can have rather cost driving effects on the MHI as a whole.

Further, the extent of driving factors for health care services and costs on the demand side - population ageing, urbanization, and social deprivation of certain groups appearing together with raising average incomes (i.e., more economic inequalities) - contributes to regional disparities in consumption and costs of health care services. Strategies to counter such driving factors could be family-friendly tax policies or investments in incentives favoring the integration of young people and foreigners. Many other political and social areas would benefit from this active strategy against problems of 'modern and globalized societies', such as social isolation, poverty, unemployment, or educational and language deficits. In addition to the driving effects of such societal phenomena, it is particularly important to remind the political authorities about the danger of possible under-utilization of outpatient health care services [43] by vulnerable groups like unemployed persons.

As only a few financing elements with potential effects on consumption and costs of health care services were modeled, the findings here are rather limited. Interestingly, however, higher deductibles accompany more intense per capita utilization of GP, specialists, hospital outpatient services, and nursing homes. It would be fruitful to investigate this finding in more detail, but to do so it would be necessary to work with data sets on the level of individuals (i.e., insured persons or patients). It seems quite plausible that one would find out that people with higher deductibles are not actually consuming more MHI services. It would be more likely that in cantons with high MHI costs - caused largely by the 'few' sick persons with normal CHF 300 deductibles - more 'healthy' people choose high deductibles in order to reduce their premium burden. That is one important reason why high (or low) consumption of MHI services and higher (or lower) deductibles co-exist in Switzerland. Moreover, the overall effects of higher deductibles on MHI services consumption should be confronted with their negative solidarity effect on chronically ill people.

The new and strong points in this work are as follows: disparities in health care utilization in space and time are investigated on a rather detailed level - quantity indicators for six individual service groups within MHI. At the same time, the variation in these detailed variables is tested with a set of influence factors that represent complete explanatory models containing supply-side, demand-side, financing-side and political-cultural variables. The models capture much variation of the dependent variables with a good overall significance in the selected explanation factors. The testing of a constant set of 12 explanation variables across the six models allows a double-way interpretation of the results: in addition to understanding how each of the six dependent variables is influenced by a set of independent variables, one can learn more about how each individual influence factor drives up or slows down all six health service groups.

What was just called a 'strong point' is simultaneously the main limitation of the study: working with quantity indicators for individual service groups within the MHI means being still on a too highly-aggregated level of modeling. When counting and explaining the number of consultations in the outpatient sector and the number of days in the inpatient sector, the changes in medical practice are not taken into account. A closer look at more detailed groups of health service providers, like different types of hospitals or different types of outpatient specialists, would have been possible, but DPS contains information only on those people who actually sent their bills to their health insurance provider. Thus, bills that were not sent for reasons such as a high deductible are not included in DPS. The actual amount of such 'hidden' MHI services and costs and their potential to bias the generated results are not known.

It is important to note that the missing quantity indicator for consumed drugs (MED) is a minor problem. As prices for MHI-remunerated drugs are determined by federal authorities on the national level, disparities in drug cost volumes can be caused only by differences in consumed quantities or a varying medical use of medicines [51].

In respect to the spatial dimension, analyses of variations on the most detailed regional level - these could be districts or even municipalities in Switzerland - would be productive. However, as mentioned in the other studies, the lack of disposable data does not allow for going below the cantonal level.

All attempts to overcome these limitations would remain limited as long as it is impossible to use individual patient data. Primarily for data protection reasons, DPS only groups patients by age, gender, and canton. While all the averages of these groups used throughout the work are normal distributed, it is well known that the individual observations are not. To underline this problem, the finding of the study that estimates that 30% of all insured people cause around 80% of total MHI costs in Switzerland [45] can be repeated. As long as such individual data sources on health care service utilization are unavailable in Switzerland, the possibilities of further validating the findings of this work remain limited.

## Appendix

See Additional file 1 for Tables 4 and 5.

## Abbreviations

MHI: Mandatory Health Insurance; GP: General Practitioners; FEM: Fixed-effects Models; REM: Random-effects Models; PRN: Pooled-regression Models; DPS: Datenpool Santésuisse; ESPOP: Swiss Population Statistics; FSO: Swiss Federal Statistical Office; MSH: Swiss Medical Statistics of Hospitals; AVAM: Labor Placement and Statistics System; SECO: Swiss State Secretariat for Economic Affairs; VZ 2000: Swiss population census 2000; CHF: Swiss francs.

## Competing interests

The author declares that he has no competing interests.

## Authors' contributions

PA. Camenzind carried out the conception and design of the study, performed the analysis of data and drafted prior versions of the article. He also read and approved the final version of the manuscript.

## Author's details

Deputy Director of Swiss Health Observatory and Ph.D. student at the University of Neuchâtel, Espace de l'Europe 10, 2010 Neuchâtel, Switzerland.

## Pre-publication history

The pre-publication history for this paper can be accessed here:

http://www.biomedcentral.com/1472-6963/12/62/prepub

## Supplementary Material

Additional file 1 **Appendix**. Tables 4 and 5. Click here for file
