# Supplementary material for: Explaining regional variations in health care utilization between Swiss cantons using panel econometric models
Source: BMC Health Serv Res. 2012 Mar 13;12:62. doi: 10.1186/1472-6963-12-62 (PMC3386862; doi:10.1186/1472-6963-12-62)
Supplement: Additional file 1 — Appendix. Tables 4 and 5. [file 1472-6963-12-62-S1.DOC]

Explaining regional variations in health care utilization between Swiss cantons using panel econometric models: Appendix

| Paul A Camenzind,[^Aff1^](#Aff1) Corresponding Affiliation: [Aff1](#Aff1) **Email**: paul.camenzind@bfs.admin.ch | |
| --- | --- |
|  | |
| Aff1 | Swiss Health Observatory and University of Neuchâtel, Espace de l’Europe 10, 2010 Neuchâtel, Switzerland |

**Appendix**

**Table 4** Swiss cantons: overview

| Name of cantons | Acronyms | Surface area^1^ in km^2^ | Population in mio. persons^2^ (2009) | Population density (persons per km^2^) | Resources index^3^ 2004/2005 | MHI costs per capita^4^ 2007 |
| --- | --- | --- | --- | --- | --- | --- |
| Aargau | AG | 1'404 | 0.600 | 427 | 87.8 | 2'167 |
| Appenzell Innerrhoden | AI | 173 | 0.016 | 93 | 82.7 | 1561 |
| Appenzell Ausserrhoden | AR | 243 | 0.053 | 218 | 79.8 | 1'869 |
| Bern | BE | 5'959 | 0.974 | 163 | 74.0 | 2'663 |
| Basel-Landschaft | BL | 518 | 0.273 | 363 | 110.2 | 2'508 |
| Basel-Stadt | BS | 37 | 0.188 | 7'378 | 148.6 | 3'291 |
| Fribourg | FR | 1'671 | 0.273 | 163 | 74.9 | 2'286 |
| Geneva | GE | 282 | 0.453 | 1'604 | 155.4 | 3'238 |
| Glarus | GL | 685 | 0.039 | 57 | 96.1 | 2'032 |
| Graubünden | GR | 7'105 | 0.192 | 27 | 84.9 | 2'141 |
| Jura | JU | 839 | 0.070 | 83 | 66.5 | 2'450 |
| Lucerne | LU | 1'493 | 0.373 | 250 | 77.0 | 2'026 |
| Neuchâtel | NE | 803 | 0.172 | 214 | 91.0 | 2'577 |
| Nidwalden | NW | 276 | 0.041 | 149 | 124.6 | 1'862 |
| Obwalden | OW | 491 | 0.035 | 71 | 67.0 | 1'937 |
| St. Gallen | SG | 2'026 | 0.475 | 235 | 77.0 | 1'993 |
| Schaffhausen | SH | 298 | 0.076 | 255 | 92.9 | 2'301 |
| Schwyz | SZ | 790 | 0.145 | 183 | 75.8 | 2'381 |
| Solothurn | SO | 908 | 0.253 | 279 | 135.6 | 2'027 |
| Thurgau | TG | 991 | 0.245 | 247 | 76.5 | 2'070 |
| Ticino | TI | 2'812 | 0.336 | 119 | 102.8 | 2'892 |
| Uri | UR | 1'077 | 0.035 | 33 | 67.0 | 1'991 |
| Vaud | VD | 3'212 | 0.702 | 219 | 96.7 | 2'777 |
| Valais | VS | 5'224 | 0.307 | 59 | 61.6 | 2'216 |
| Zug | ZG | 239 | 0.111 | 465 | 204.0 | 2'019 |
| Zurich | ZH | 1'729 | 1.351 | 781 | 132.1 | 2'381 |
| **Switzerland** | **CH** | **41'285** | **7.786** | **189** | **100.0** | **2'443** |

1) Source: [[35](#CR35)].

2) Source: [[34](#CR34)].

3) The resources index is an indicator for the financial power of a canton calculated from the amount of tax revenues per head of the cantonal population. (see Eidgenössische Finanzverwaltung, Verordnung vom 7.11.2007 über den Finanz- und Lastenausgleich (FiLaV, SR 613.21, <http://www.admin.ch/ch/d/sr/6/613.21.de.pdf>).

4) Source: [[33](#CR8)].

**Table 5** Dependent and independent variables

| **Variables tested** | **Acronym** | **Sources^1)^** **(Institution)** | **Years** | **Definition** |
| --- | --- | --- | --- | --- |
| **Dependent Variables** |  |  |  |  |
| General practitioners (GP): basic services | **AZG** | DPS / ESPOP (santésuisse / FSO) | 2000 – 2007 | MHI basic services^2)^ (GP)per head of population^3)^ |
| Specialist doctors: basic services | **AZS** | DPS / ESPOP (santésuisse / FSO) | 2000 – 2007 | MHI basic services (specialists)per head of population |
| Hospital inpatient: hospital days | **HOS** | DPS / ESPOP (santésuisse / FSO) | 2000 – 2007 | MHI hospital days (inpatient) per head of population |
| Hospital outpatient: consultations | **AMB** | DPS / ESPOP (santésuisse / FSO) | 2000 – 2007 | MHI consultations (outpatient)per head of population |
| Drugs outpatient: costs | **MED** | DPS / ESPOP (santésuisse / FSO) | 2000 – 2007 | MHI costs^4)^ for drugs (outpatient)per head of population in CHF |
| Nursing homes: days of stay | **SOM** | DPS / ESPOP (santésuisse / FSO) | 2000 – 2007 | MHI days of care (nursing homes)^5)^ per head of population |
| **Independent Variables** |  |  |  |  |
| **Supply-side** |  |  |  |  |
| Density of general practitioners (GP) | **GRU** | DPS / ESPOP (santésuisse / FSO) | 2000 – 2007 | Number of GP (equivalents full-time) per 100,000 population |
| Density of specialists (including psychiatrists) | **SPZ** | DPS / ESPOP (santésuisse / FSO) | 2000 – 2007 | Number of specialists (equivalents full-time) per 100,000 population |
| Density of hospital beds | **BED** | MSH / ESPOP (FSO) | 2000 – 2007 | Number of beds (K11 +12 +21 +22 +23) per 100,000 population |
| Share of hospital outpatient costs | **PAM** | DPS (santésuisse) | 2000 – 2007 | MHI share of costs hospital outpatient on total costs outpatient CHI |
| **Demand-side** |  |  |  |  |
| Population 65+/85+ | **ALT65 / ALT85** | ESPOP (FSO) | 2000 – 2007 | Share of people 65+ / 85+ years per 1,000 population |
| Population density | **POP** | Areal statistics / ESPOP (FSO) | 2000 – 2007 | Number of inhabitants per canton (persons per hectare) |
| Unemployment rate | **ALQ** | AVAM / ESPOP (SECO / FSO) | 2000 – 2007 | Registered unemployed persons(Ø year) per 1,000 population |
| Average cantonal income^6)^ | **VEL** | Statistics on National Accounts (FSO) | 1998 **–** 2005 | Log. transform of cantonal income^7)^ per head of population in CHF |
| **Financing** |  |  |  |  |
| Higher deductibles | **FRA** | DPS (santésuisse) | 2000 – 2007 | MHI share of insured with deductible > CHF 300 on total of 1,000 insured |
| Alternative MHI-plans | **MOD** | DPS (santésuisse) | 2000 – 2007 | MHI share of insured alternative plans on total of 1,000 insured |
| **Politics / various** |  |  |  |  |
| Latin-speaking population | **LAT** | VZ 2000 (FSO) | 2000 | Share of primary language ‘non-German’ per 1,000 population |
| Trend variable (linear) | **TRD** | - | 2000 – 2007 | 2000 = 1, 2001 = 2, etc. |

1) Sources: [[3](#CR3),33-[38](#CR38)]

2) Basic services = sum of consultations and home visits (no drugs).

3) Residence rule: utilization of providers located in the same canton or of providers located in other cantons by the resident population of the canton.

4) Drugs delivered by outpatient physicians and pharmacies.

5) Total social-medical institutions (for elderly, chronically ill, and disabled people).

6) After checking the distributional characteristics of all dependent and independent variables, it was decided to transform the average cantonal income per capita VEL into its logarithmic form.

7) Per capita sum of income that residential units (private households, firms, public households) of a canton receive for their productive activity for the economy inside or outside the canton.
